# Oxidative Stress Induced by the Deubiquitinase Inhibitor b-AP15 Is Associated with Mitochondrial Impairment

**DOI:** 10.1155/2019/1659468

**Published:** 2019-06-10

**Authors:** Xiaonan Zhang, Belén Espinosa, Amir Ata Saei, Padraig D'Arcy, Roman A. Zubarev, Stig Linder

**Affiliations:** ^1^Department of Oncology-Pathology, Karolinska Institutet, SE-17176 Stockholm, Sweden; ^2^Division of Biochemistry, Department of Medical Biochemistry and Biophysics, Karolinska Institute, SE-17177 Stockholm, Sweden; ^3^Department of Medical Biochemistry and Biophysics, Division of Physiological Chemistry I, Karolinska Institute, SE-17177 Stockholm, Sweden; ^4^Division of Drug Research, Department of Medical and Health Sciences, Linköping University, SE-58183 Linköping, Sweden

## Abstract

Inhibitors of the 20S proteasome such as bortezomib are cytotoxic to tumor cells and have been proven to be valuable for the clinical management of multiple myeloma. The therapeutic efficacy of bortezomib is, however, hampered by the emergence of acquired resistance. Available data suggest that blocking proteasome activity at the level of proteasome-associated deubiquitinases (DUBs) provides a mechanism to overcome resistance to bortezomib and also to other cancer therapies. The small molecule b-AP15 is an inhibitor of proteasome-associated DUB activity that induces both proteotoxic stress and increases in the levels of reactive oxygen species (ROS) in tumor cells. Antioxidants have been shown to decrease apoptosis induction by b-AP15 and we here addressed the question of the mechanism of redox perturbation by this compound. We show that oxidative stress induction by b-AP15 is abrogated in cells deprived of mitochondrial DNA (*ρ* ^0^ cells). We also show associations between the level of proteotoxic stress, the degree of mitochondrial dysfunction, and the extent of induction of hemeoxygenase-1 (HO-1), a target of the redox-regulated Nrf-2 transcription factor. Decreased expression of COX5b (cytochrome c oxidase subunit 5b) and TOMM34 (translocase of outer mitochondrial membrane 34) was observed in b-AP15-treated cells. These findings suggest a mitochondrial origin of the increased levels of ROS observed in cells exposed to the DUB inhibitor b-AP15.

## 1. Introduction

Aberrant accumulation of misfolded or damaged proteins is associated with reduced cell survival [1]. Protein quality control is primarily mediated by the ubiquitin-proteasome system (UPS), the major eukaryotic proteolytic pathway, and is essential for cell viability [2, 3]. The proteasome degrades the bulk of cellular protein and is instrumental to the regulation of essential cellular processes such as cell cycle progression [4–7]. Misfolded, damaged, or temporally regulated proteins are marked for removal by the destruction tag ubiquitin that signals traffic to the proteasome for degradation. Once at the proteasome, ubiquitin is removed from polyubiquitinated proteins by deubiquitinases (DUBs) localized in the 19S regulatory particle, to facilitate translocation into the 20S core particle where degradation takes place [8, 9]. Cancer cells, characterized by rapid protein synthesis and unlimited proliferation, face an extreme load of misfolded proteins and therefore have an increased requirement for UPS-mediated protein turnover [10, 11]. Under conditions of proteasome inhibition, misfolded proteins accumulate in tumor cells, resulting in pleiotropic effects such as induction of cytosolic chaperones, endoplasmic reticulum (ER) stress, and oxidative stress [12–14].

Inhibitors of the 20S proteasome such as bortezomib and carfilzomib are used for treatment of multiple myeloma and have changed the clinical course of this disease [15, 16]. However, both intrinsic and acquired resistance to bortezomib limit its therapeutic efficacy [17]. A number of different mechanisms have been described to result in bortezomib resistance, including mutations in the PSMB5 subunit and overexpression of this subunit [17].

The redox state is important for cell survival, proliferation, and apoptosis [18]. Reactive oxygen species (ROS) may be harmful to cells leading to oxidative damage such as lipid peroxidation but may also be second messengers controlling signaling pathways [19]. The three major sources of ROS in the cell are mitochondria, peroxisomes, and the endoplasmic reticulum (ER) [20, 21]. Oxidative stress has been described to be induced by proteasome inhibitors [13, 14, 22], and antioxidants have been shown to decrease the apoptotic effects of these drugs [12, 13]. Oxidative stress resulting from proteasome inhibition has been attributed to ER stress [23] and to mitochondrial dysfunction [13] by various investigators.

We and the others have shown that the dienone compounds b-AP15, VLX1570, and RA-9 inhibit the activities of proteasome-associated DUBs [24–27], in particular USP14 [27]. This class of compounds induce apoptosis in tumor cells defective in TP53 [28] and overexpressing BCL2 [24, 29]. The ability of these compounds to selectively kill tumor cells while being largely insensitive to TP53 mutational status and defects in apoptotic machinery is interesting both from a mechanistic and therapeutic point of view, in particular considering their anticancer activities *in vitro* and in animal models [24, 25, 27, 29–41]. Interestingly, b-AP15 shows antiproliferative activity on myeloma cells resistant to bortezomib [33] and melanoma cells resistant to MAPK-targeting therapies [27]. We recently showed that the strong proteotoxicity induced by b-AP15 resulted in mitochondrial toxicity [42]. We and the others have shown that b-AP15 induces reactive oxygen species in tumor cells and that antioxidants decrease the apoptotic response [22, 27, 43]. These findings prompted us to examine whether oxidative stress induction by b-AP15 is mechanistically linked to mitochondrial dysfunction. We here provide experimental evidence in support of this notion.

## 2. Material and Methods

### 2.1. Chemicals and Antibodies

b-AP15 was obtained from OnTarget Chemistry (Uppsala, Sweden), Velcade (bortezomib, Selleck Chem) and CpdA [44] from Novartis. Antibodies used were anti-actin (Sigma-Aldrich catalogue number A5441), anti-Ub-K48 (Merck Millipore catalogue number 05-1307), anti-HMOX (BD Biosciences catalogue number 610713), anti-Hsp60 (Cell Signaling catalogue number 12165), anti-HSP40 (Cell Signaling catalogue number 4868), anti-Nrf-2 (Cell Signaling catalogue number 12721), anti-CHOP (Cell Signaling, catalogue number 5554), anti-HSP70B′ (Abcam catalogue number ab69408), and anti-MTCOXII2 (Abcam catalogue number ab110258).

### 2.2. Cell Culture and Drug Treatment

HCT116 colon carcinoma cells were maintained in McCoy's 5A modified medium with 10% FBS and 1% penicillin. HeLa cells were cultured in DMEM medium with supplemented 10% FBS and 1% penicillin. Cell lines were used at low passage numbers and checked for absence of mycoplasma. Drugs were dissolved in DMSO for final concentrations of DMSO 0.5%. CpdA was used at a concentration of 10 *μ*M as described previously [45].

### 2.3. Western Blot Analysis

Cell extract proteins were resolved by 3-8% Tris-Acetate protein gels (Invitrogen, Carlsbad, CA) to detect polyubiquitinated proteins and 4-12% Bis-Tris protein gels to detect other proteins mentioned in the text, then transferred onto a PVDF membrane for western blotting [46]. Blots were developed by enhanced chemiluminescence (Amersham, Arlington Heights, IL).

### 2.4. Electron Microscopy

Cells were treated with b-AP15 for different times and fixed with 2.5% glutaraldehyde. Cells were postfixed in 1% osmium tetroxide, dehydrated, and embedded in epoxy resin. Ultrathin sections were prepared for analysis in a transmission electron microscope. Electron microscopy was performed by Kjell Hultenby at the Department of Laboratory Medicine, Clinical Research Center, Karolinska Universitetssjukhuset Huddinge, Sweden.

### 2.5. Glutathione Assays

For measurement of glutathione, cells were treated with 1.0 *μ*M b-AP15 for 6 h. Cells were collected and concentrations of GSSG and total glutathione (GSH + GSSG) were analyzed using the quantification kit for oxidized and reduced glutathione (#38185, Sigma) as described. The final concentration of GSH was determined by equation of GSH = total glutathione (GSH + GSSG)–GSSG × 2. The data was analyzed using GraphPad Prism 7.

### 2.6. Measurements of G6PD, Glutathione Peroxidase, and Malondialdehyde

Drug-treated cells were washed with PBS and frozen at -80°C as cell pellets. Pellets were shipped to Biochemikon SAS, 94000 Créteil, France (study director Marc Conti). Cell pellets were sonicated, and enzymatic activities and substrate concentration measurements were performed. Glutathione peroxidase activity was measured according to Paglia and Valentine [47]. G6PD activity was adapted/optimized from Beutler [48]. MDA measurements were determined according to Conti et al. [49].

### 2.7. Measurements of Oxygen Consumption

OCR (oxygen consumption rates) were measured using a Seahorse XF24 extracellular flux analyzer in real time as recommended by the manufacturer (Seahorse Bioscience, North Billerica, MA, USA). Cells (60,000 cells/well) were plated in 100 *μ*L culture medium in XF24-well cell plates with blank control wells. Prior to the measurements, the medium was replaced with 500 *μ*L Seahorse assay media (1 mM pyruvate, 25 mM glucose, and 2 mM glutamine) at 37°C without CO_2_ for 1 h.

### 2.8. Generation of HeLa Rho^0^ (*ρ* ^0^) Cells

Hela cells were grown in DMEM medium supplemented with 100 ng/mL EtBr and 50 *μ*g/mL uridine [50]. DNA was isolated using PureLink® Genomic DNA Mini Kit (Thermo Fisher Scientific), and mtDNA and nDNA were amplified by Human Mitochondrial DNA (mtDNA) Monitoring Primer Set (Takara). Copy number was measured using a 7500/7500 Fast Real-Time PCR System (Applied Biosystems), and mitochondria DNA to nuclear DNA ratios were calculated by the program supported by Takara. The absence of mtDNA-encoded protein MTCOXII2 in Rho^0^ cells was confirmed by immune blotting.

### 2.9. Isolation of Mitochondria

Mitochondrial isolation process was performed as [42].

### 2.10. Proteomics

Proteomic analysis was performed as described [42]. The raw data from LC-MS were analyzed by MaxQuant, version 1.5.6.5 [51]. The Andromeda search engine [52] searched MS/MS data against the International Protein Index (human, version UP000005640_9606, 92957 entries). Protein abundances were normalized by the total protein abundance in each sample. Mitochondrial proteins were further selected from total detected protein pool using MitoCarta (http://www.broad.mit.edu/publications/MitoCarta) supplied by [53].

### 2.11. Statistical Analysis

Statistical significance was evaluated by Student's two-tailed paired *t*-test (parametric) or Mann-Whitney *U* test (nonparametric). Protein expression data were compared using Spearman correlation coefficients.

## 3. Results

### 3.1. The Deubiquitinase Inhibitor b-AP15 Affects Mitochondrial Structure and Function

**Figure 1:**
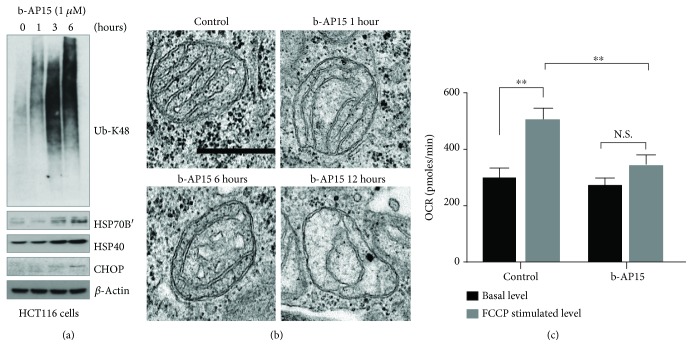
Induction of mitochondrial dysfunction in HCT116 cells by the deubiquitinase inhibitor b-AP15. (a) HCT116 cells were exposed to 0.5% DMSO or 1 *μ*M b-AP15 for 1, 3, and 6 hours, and extracts were prepared and subjected to immunoblotting using the indicated antibodies. (b) Electron micrographs of HCT116 cells treated with b-AP15 for 1, 6, and 12 h. Scale bar = 0.5 *μ*m. (c) Basal and maximal oxygen consumption rates (OCR) were measured after a 5-hour exposure of HCT116 cells to 1 *μ*M b-AP15 using a Seahorse XF analyzer. Uncoupled respiration was measured after exposure to carbonyl cyanide-4-(trifluoromethoxy)-phenylhydrazone (FCCP) (mean ± S.D.; ∗∗*p* < 0.01).

We have previously reported that b-AP15, an inhibitor of proteasome-associated deubiquitinases, generates both proteotoxic stress and oxidative stress [22, 24, 26, 27] and also induces mitochondrial dysfunction [42]. As shown in Figure 1(a), treatment of HCT116 cells with 1 *μ*M b-AP15 resulted in increased levels of K48-linked polyubiquitin conjugates and induction of the chaperones HSP70B′ and HSP40 as well as the ER marker CCAAT-enhancer-binding protein homologous protein (CHOP) [54, 55]. Consistent with previous results, mitochondria became increasingly deformed during exposure to b-AP15 (Figure 1(b)). Mitochondrial function was examined by monitoring oxygen consumption rates using a Seahorse XF24 analyzer. Confirming previous results [42], the stimulation of oxygen consumption by carbonyl cyanide p-trifluoromethoxy-phenylhydrazone (FCCP) was reduced in b-AP15-exposed cells (Figure 1(c)), showing a decrease in maximal respiration capacity.

### 3.2. b-AP15 Induces Oxidative Stress but Not Lipid Peroxidation

**Figure 2:**
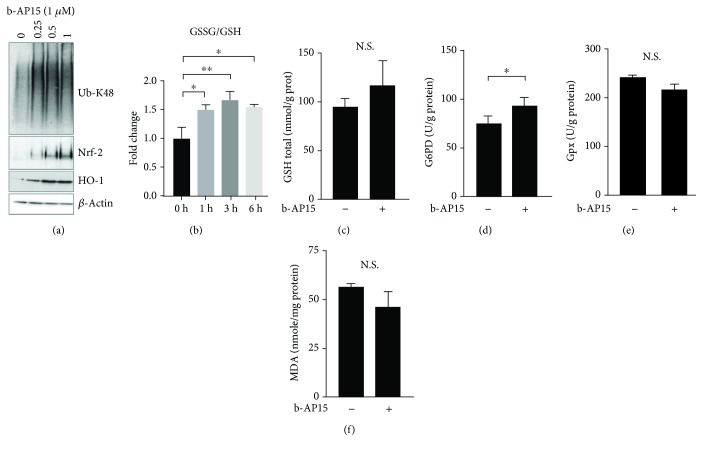
Evidence of oxidative stress in b-AP15-exposed HCT116 cells. (a) HCT116 cells were exposed to 0.5% DMSO or b-AP15 (0.25, 0.5, and 1.0 *μ*M in 0.5% DMSO) for 6 h, and extracts were prepared and subjected to immunoblotting using the indicated antibodies. (b) Increases in the ratio of GSSG/GSH in HCT116 cells exposed to 1 *μ*M b-AP15 for the indicated times (mean ± S.D.; ∗*p* < 0.05, ∗∗*p* < 0.01; *n* = 3). (c) Total levels of GSH were determined in vehicle-treated cells and in cells exposed to 1 *μ*M b-AP15 for 6 h (mean ± S.D.; *n* = 3; N.S.: not significant at *p* < 0.05). (d) Glucose-6-phosphate dehydrogenase activity in HCT116 cells exposed to 1 *μ*M b-AP15 for 6 h compared to vehicle-treated cells (mean ± S.D.; ∗*p* < 0.05; *n* = 3). (e) Glutathione peroxidase activity in HCT116 cells exposed to 1 *μ*M b-AP15 for 6 h compared to vehicle-treated cells (mean ± S.D.; *n* = 3). (f) Malondialdehyde levels in HCT116 cells exposed to 1 *μ*M b-AP15 or vehicle for 6 h. Statistical significance was calculated using Student's *t*-test in (b)–(f).

Previous studies demonstrated increased levels of intracellular ROS in b-AP15-treated HCT116 cells [22] and in melanoma cells [27]. Exposure of HCT116 cells to b-AP15 resulted in increased levels of the redox-regulated transcription factor Nrf-2 (nuclear factor erythroid 2-related factor 2) and its downstream target HO-1. This increases occurred at doses that induced the accumulation of high molecular weight K48-linked polyubiquitin conjugates (Figure 2(a)). Furthermore, a significant increase in the GSSG/GSH ratio was observed in b-AP15-exposed HCT116 cells, whereas no significant increases in total GSH levels were observed (Figures 2(b) and 2(c)). Increased glucose 6-phosphate dehydrogenase (G6PD) enzyme activity, the rate-limiting enzyme of the pentose phosphate pathway, was observed in b-AP15-treated HCT116 cells (*p* = 0.015) (Figure 2(d)). In contrast, glutathione peroxidase (GPx) activity levels were not significantly altered by b-AP15 treatment (Figure 2(e)). Malondialdehyde is a product of lipid peroxidation and a marker of oxidative damage [56]. Increased levels of malondialdehyde were not observed in HCT116 cells exposed to b-AP15 for 6 h (Figure 2(f)).

### 3.3. Induction of Oxidative Stress Is Dependent on Functional Mitochondria

**Figure 3:**
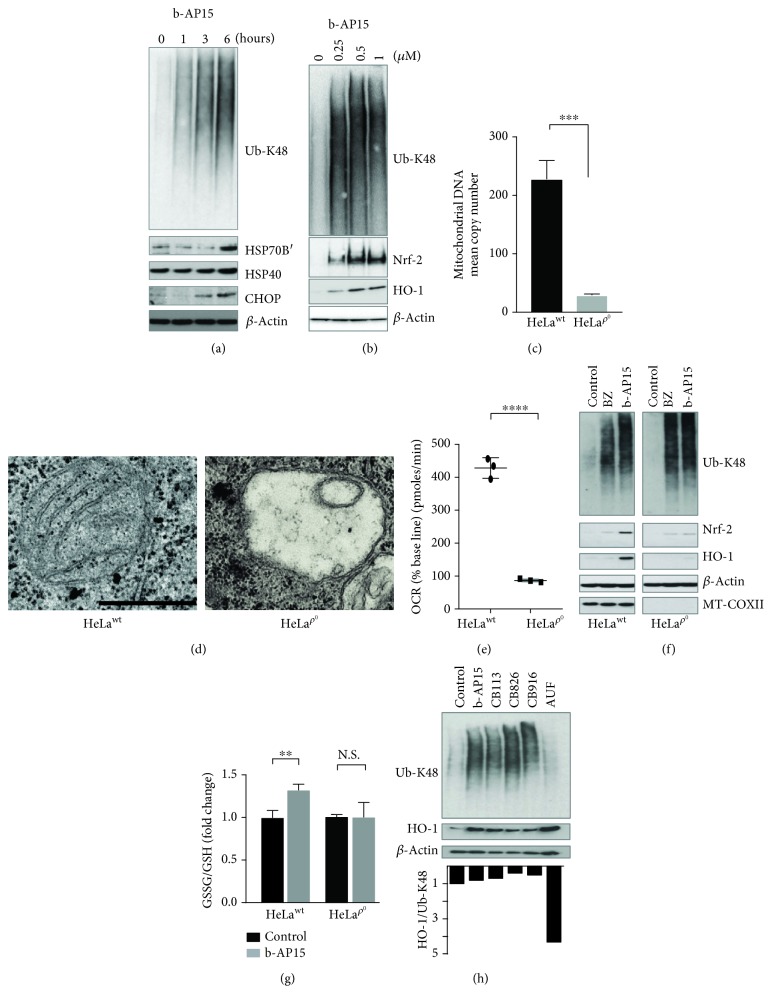
HeLa Rho^0^ (*ρ* ^0^) cells show a decreased oxidative stress response to b-AP15. (a) HeLa cells were exposed to 0.5% DMSO or 1 *μ*M b-AP15 for 1, 3, and 6 hours, and extracts were prepared and subjected to immunoblotting using the indicated antibodies. All cultures received 0.5% DMSO. (b) HeLa cells were exposed to 0.5% DMSO or b-AP15 (0.25, 0.5, and 1.0 *μ*M in 0.5% DMSO) for 6 h, and extracts were prepared and subjected to immunoblotting using the indicated antibodies. (c) HeLa cells were exposed to EtBr and uridine to generate mitochondrial DNA depleted cells (HeLa *ρ* ^0^). The ratio of mtDNA to nDNA was compared in HeLa parental and *ρ* ^0^ cells using RT-PCR (∗∗∗*p* < 0.001). (d) Electron micrographs of mitochondria in HeLa parental and *ρ* ^0^ cells. Scale bar = 0.5 *μ*m. (e) Basal oxygen consumption rates (OCR) of HeLa parental and *ρ* ^0^ cells (*n* = 3; mean ± S.D.; ∗∗∗∗*p* < 0.0001). (f) HeLa *ρ* ^0^ cells were treated with 100 nM bortezomib (BZ) or 1*μ*M b-AP15 for 5 h followed by western blot analysis for K48-linked polyubiquitin chains, Nrf-2, HO-1, MT-COXII, and *β*-actin. Note the impaired induction of Nrf-2 and HO-1 by UPS inhibitors in *ρ* ^0^ cells. (g) The ratio of GSSG/GSH was determined in parental HeLa and *ρ* ^0^ cells exposed to 1 *μ*M b-AP15 or vehicle for 6 h (*n* = 3; mean ± S.D.; ∗∗*p* < 0.01). (h) HCT116 cells were exposed to 0.5% DMSO, 1 *μ*M b-AP15, 5 *μ*M CB113, 5 *μ*M CB826, 5 *μ*M CB916, and 1.5 *μ*M auranofin (AUF) for 6 h, and extracts were prepared and subjected to immunoblotting using the indicated antibodies.

Attempts to generate HCT116 cells deficient in mitochondrial DNA (*ρ* ^0^ cells) by exposure to ethidium bromide were unsuccessful (not shown), possibly due to HCT116 cells being dependent on oxidative phosphorylation [57]. We therefore used HeLa cells, for which *ρ* ^0^ derivatives have been described [58]. Similar to the response in HCT116 cells, increases in polyubiquitinated proteins, chaperones, and CHOP were observed in HeLa cells exposed to b-AP15 (Figure 3(a)) and b-AP15 induces an apoptotic response in HeLa cells (Supplementary Fig.  1). Furthermore, Nrf-2 and HO-1 induction was observed also in b-AP15-treated HeLa cells (Figure 3(b)). Continuous exposure of HeLa cells to low doses of ethidium bromide resulted in cells with a reduced copy number of mitochondrial DNA (Figure 3(c)), aberrant mitochondrial morphology (Figure 3(d)), low oxygen consumption rates (Figure 3(e)), and no detectable expression of the mitochondria genome-encoded protein MTCOXII (Figure 3(f)). Exposure of HeLa *ρ* ^0^ cells to b-AP15 resulted in a dramatic abrogation of Nrf-2 and HO-1 induction (Figure 3(f)). Furthermore, and in contrast to the response observed in HeLa parental cells, exposure of HeLa *ρ* ^0^ cells to b-AP15 did not result in an increased GSSG/GSH ratio (Figure 3(g)). These findings are consistent with the notion that oxidative stress induction by b-AP15 is dependent on functional mitochondria. We considered the possibility of decreased levels of protein synthesis in *ρ* ^0^ cells, resulting in reduced proteotoxic stress and, as a consequence, lower oxidative stress. However, the levels of polyubiquitinated proteins induced by b-AP15 or bortezomib were comparable in HeLa parental and *ρ* ^0^ cells (Figure 3(f)).

Our observations suggest an association between oxidative stress and mitochondria perturbation as a result of proteotoxic stress generated by b-AP15. One alternative mechanism of oxidative stress induction is inhibition of thioredoxin reductase (TrxR) activity, previously shown for b-AP15 [43]. To examine this possibility, we used a number of recently identified inhibitors of proteasome-associated DUBs that do not inhibit TrxR (Supplementary Fig.  2). We found that three different and chemically unrelated molecules that do not inhibit TrxR all induced the expression of the Nrf-2 target HO-1 (Figure 3(h)). Auranofin, a well-documented inhibitor of TrxR [59], induced HO-1 expression but did not induce accumulation of polyubiquitinated proteins (Figure 3(h)). These findings show that induction of the Nrf-2 target protein HO-1 by inhibitors of proteasome-associated DUBs does not require inhibition of TrxR.

### 3.4. Increased Levels of Proteotoxic Stress Result in Decreased Oxygen Consumption and Increased Expression of HO-1

**Figure 4:**
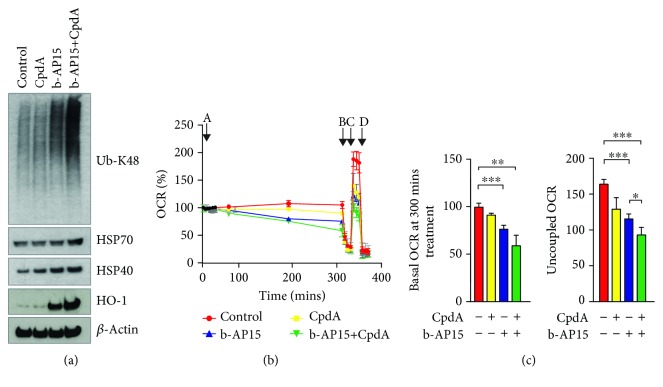
Increased levels of proteotoxic stress are associated with decreased mitochondrial function and increased induction of HO-1. (a) HCT116 cells were exposed to 0.5% DMSO, 1 *μ*M b-AP15, and 10 *μ*M CpdA for 6 h, as indicated. Extracts were prepared and subjected to immunoblotting using the indicated antibodies. Note the increased levels of polyubiquitinated proteins, Hsp70, and HO-1 in cells exposed to b-AP15 and the ER translocation inhibitor CpdA. (b, c) HCT116 cells were treated with b-AP15 (1 *μ*M) and/or CpdA (10 *μ*M) for 5 hours and oxygen consumption rates were measured using a Seahorse XF analyzer (*n* = 3 in each group). A: DMSO or compounds; B: oligomycin; C: FCCP; D: antimycin and rotenone. (b) Measurement of OCR in real time after exposure to different compounds; (c) *left*: basal OCR after 300 min of treatment with compounds (mean ± S.D.; ∗∗∗*p* < 0.0001; *n* = 3); *right*: uncoupled OCR after addition of FCCP (mean ± S.D.; ∗∗∗*p* < 0.0001; ∗*p* < 0.05; *n* = 3).

Further increases in the levels of proteotoxic stress are expected to result in increased mitochondrial damage and elevated oxidative stress. We used the CpdA, an inhibitor of Sec61-mediated anterograde protein translocation over the ER membrane [44], to test this prediction. Consistent with previous results [45], cotreatment of b-AP15 with CpdA induced strong accumulation of polyubiquitinated proteins and overexpression of HSP70 and HSP40 chaperones in HCT116 cells (Figure 4(a)). Cotreatment resulted in stronger reductions in oxygen consumption rates compared to treatments with b-AP15 or CpdA alone (Figure 4(b)). Cotreatment with b-AP15 and CpdA also resulted in stronger increases in HO-1 (Figure 4(a)), consistent with the notion of an association between proteotoxic stress and oxidative stress.

### 3.5. Alterations of the Mitochondrial Proteome Reveal Decreased Expression of COX5b

**Figure 5:**
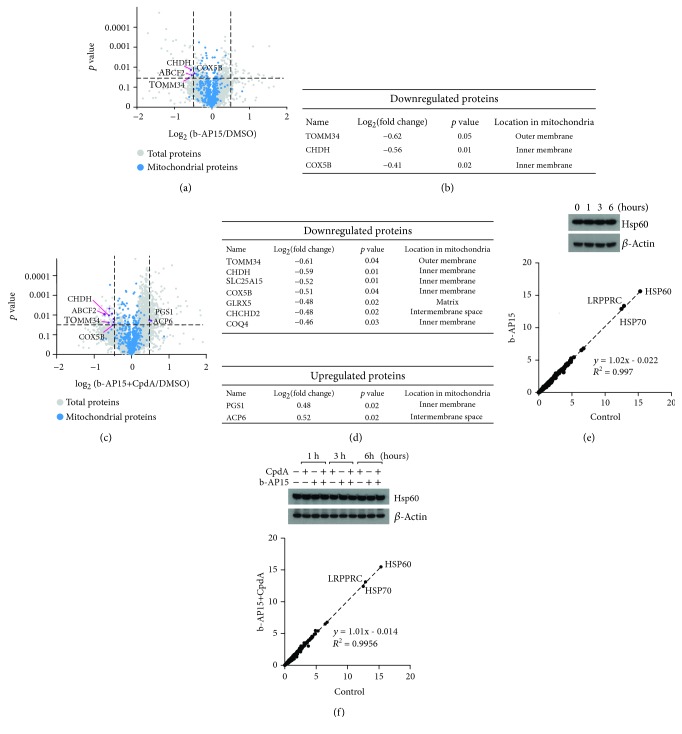
Proteomic analysis of mitochondrial proteins. (a) Volcano plot showing log_2_(fold change) versus *p* values for proteins from isolated mitochondria prepared from HCT116 cells treated with DMSO or 1 *μ*M b-AP15 for 6 h. (b) Top candidates with significant changes from (a) (*p* ≤ 0.05, log_2_ ≥ 0.4 or log_2_ < −0.4). (c) Volcano plot showing log_2_(fold change) versus *p* values for proteins from isolated mitochondria prepared from HCT116 cells treated with DMSO or 1 *μ*M b-AP15 and 10 *μ*M CpdA for 6 h. (d) Top candidates with significant changes from (c) (*p* ≤ 0.05, log_2_ ≥ 0.4 or log_2_ < −0.4). (e, f) Upper part: HCT116 cells were exposed to 0.5% DMSO, 1 *μ*M b-AP15 in the presence or absence of 10 *μ*M CpdA for 1, 3, and 6 h, as indicated. Extracts were prepared and subjected to immunoblotting using antibodies to Hsp60 and *β*-actin. (e, f) Lower part: mitochondria were purified from cells treated with 1 *μ*M b-AP15 in the presence or absence of 10 *μ*M CpdA and analyzed by shotgun proteomics. Data was normalized to control samples (treated with 0.5% DMSO).

Damaged mitochondria in b-AP15-treated cells are not cleared by mitophagy and can be purified and analyzed by proteomics and other methods [42]. Three mitochondrial proteins were found to be significantly downregulated in mitochondrial preparations from b-AP15-treated cells: TOMM34 (translocase of outer mitochondrial membrane 34), CHDH (choline dehydrogenase), and COX5b (cytochrome c oxidase subunit 5B) (Figures 5(a) and 5(b)). Cotreatment with b-AP15 and CpdA resulted in a similar or larger decrease in the levels of these proteins and significant downregulation of some additional proteins (Figures 5(c) and 5(d)). COX5b is a component of the electron transport chain and the decrease of this protein may explain the decreases in mitochondrial oxidative phosphorylation observed in b-AP15-treated cells.

Mitochondria possess a protein folding machinery (HSP60, HSP10, TRAP1, and mtHSP70) to respond to the misfolding stress inside of mitochondria (UPR^mt^) [60]. We addressed the question of whether b-AP15 affects mitochondrial protein homeostasis, leading to induction of HSP60. However, HSP60 expression was not affected by treatment with b-AP15 in the absence or presence of CpdA (Figures 5(e) and 5(f)).

## 4. Discussion

b-AP15 and similar compounds have been shown to induce apoptotic responses in tumor cells overexpressing BCL2 family proteins and cells defective in TP53 [24, 29]. Previous reports have demonstrated induction of both strong oxidative stress and proteotoxicity by b-AP15 [22, 27, 61] and also showed evidence of mitochondrial toxicity [42]. These findings led to the hypothesis that oxidative stress induction by this class of compounds is mechanistically linked to mitochondrial dysfunction. We here found weaker induction of the Nrf-2 target HO-1 and decreased elevation of GSSG/GSH ratios in *ρ* ^0^ cells exposed to b-AP15, consistent with a mitochondrial involvement in b-AP15-induced oxidative stress. We also found that increasing the level of proteotoxic stress by inhibiting anterograde ER translocation resulted in increased induction of expression of HO-1. These findings are consistent with the hypothesis of a mitochondrial origin of the increased levels of ROS observed in cells exposed to the DUB inhibitor b-AP15.

We and the others have shown that b-AP15 induces phosphorylation of JNK and that inhibition of JNK signaling decreases the apoptotic response [22, 27]. Both JNK signaling and apoptosis are decreased by antioxidant treatment [22]. These findings point to a perturbation of the intracellular redox state being involved in induction of apoptosis. The levels of malondialdehyde, a product of lipid peroxidation of polyunsaturated fatty acids [56], did not increase during b-AP15 treatment and available data suggest that activation of antioxidant systems prevents direct oxidative damage. We here found significant increases in glucose 6-phosphate dehydrogenase (G6PD) activity, leading to a larger potential of NADPH generation [62]. The lack of detectable lipid peroxidation can be argued to mean that b-AP15 does not induce “oxidative stress” by a more stringent definition but merely induces redox imbalances. Although these imbalances are sufficient to induce Nrf-2 and phosphorylation of JNK, they appear to be contained by antioxidant defenses. It should be pointed out, however, that the lack of detectable increases in lipid peroxidation does not necessarily mean that oxidative damage to macromolecules does not occur in specific cellular compartments. For a discussion of redox perturbations, oxidative stress, and oxidative damage, see [63].

We previously presented evidence favoring that the mitochondrial damage that occurs during exposure to b-AP15 is due to the accumulation of misfolded proteins on the outer mitochondrial membrane [42]. This observation did not explain the decrease in oxidative phosphorylation that occurs during drug treatment. We here show downregulation of the COX5b protein, an essential component of cytochrome c oxidase [64]. Cytochrome c oxidase is a key enzyme in the overall regulation of cellular energy production in eukaryotes [65]. Decreases in COX5b have been associated with mitochondrial dysfunction in various conditions [66], and upregulation of COX5b has been observed in energy-demanding cell types and healthy tissues. It has also been demonstrated that downregulation of COX5b by siRNA increases mitochondrial ROS generation [67]. The levels of the yeast homologue of COX5b, COX IV-1, have been shown to be posttranscriptionally regulated by the cardiolipin content of the mitochondria [68], and COX5b has also been reported to be regulated by carbon source and oxygen [64, 69].

TOMM34 (34 kDa translocase of the outer mitochondrial membrane) was originally identified as a component of the mitochondrial import machinery for nucleus-encoded mitochondrial proteins and has been reported to form a complex with both Hsp70 and Hsp90 as a cytosolic scaffolding cochaperone [70–72]. We observed decreased levels of TOMM34 in mitochondrial preparations in parallel with elevated levels of HSP70B′. It is possible that an elevated demand of TOMM34 in assisting Hsp70/Hsp90 in different folding processes in the cytosol limits the availability of TOMM34 proteins on the outer mitochondrial membrane.

Despite its strong cytotoxicity to tumor cells, b-AP15 and similar compounds show limited activity against normal cells [3, 6] and its cytotoxicity is likely to be dependent on the elevated rate of protein turnover in tumor cells. The results presented here suggest that oxidative stress is coupled to proteotoxic stress, leading to an enhancement of the effects on proteasome inhibition. b-AP15 has shown activity in a number of tumor models, including multiple myeloma [12, 13], Ewing's carcinoma [14], Waldenström's macroglobulinaemia [15], melanoma [9], and colon cancer [3]. The *in vivo* efficacy of b-AP15 is limited by the poor solubility of the compound, and efforts are ongoing to improve the pharmacological properties of this class of molecules. If these efforts are crowned by success, inhibitors of proteasome-associated DUBs could be important drugs in an increasing arsenal of therapeutic options for cancer.

## Data Availability

The data used to support the findings of this study are included within the article.

## Supplementary Materials

Supplementary Materials Supplementary Fig. 1: induction of caspase cleavage activity by b-AP15 in HeLa cells. HeLa cells were exposed to 1 *μ*M b-AP15 for 24 hours in the presence of absence of BIK siRNA or scrambled control siRNA as indicated. Exponentially growing HeLa cells were seeded in 100 mm dishes at 106 cells per plate and grown for 24 h and then transfected with BIK siRNA (Qiagen) at a final concentration of 10 nM using Lipofectamine RNAi MAX (Invitrogen). Cells were incubated for another 72 h and then treated with DMSO or b-AP15 for another 18 h. Apoptosis was determined by the measurement of the accumulation of caspase-cleaved apoptosis product in cells and culture medium using the M30 Apoptosense ELISA assay (VLVbio, Stockholm). The assay measures caspase-cleaved keratin 18 (K18-Asp396) (Bivén et al., Apoptosis 8 (2003) 263). BIK is a BH3-only protein that has been described to be associated with oxidative stress-induced apoptosis (Bodet et al., Br J Cancer 12 (2010) 1808). Means + S.D. are shown (*n* = 3). Statistical significance was determined using Student's *t*-test. Supplementary Fig. 2: oxidative stress induced by b-AP15 is not due to the inhibition of thioredoxin reductase (TrxR) activity. CB113 (ChemBridge ID 6943113), CB826 (ChemBridge ID 6556826), or CB916 (ChemBridge ID 6237916) was added to thioredoxin reductase enzyme assay mixes at 20 *μ*M in Tris-EDTA and incubated for 5 hours. Reactions contained 50 nM rTrxR (21 U/mg), 200 *μ*M NADPH, and 1 mg/mL BSA. Reactions were started by the addition of 2.5 mM of DTNB and absorbance was read at 412 nms. The TrxR inhibitor auranofin and Juglone were used as positive controls. Note, none of these three proteasome inhibitors targeted the TrxR activity at the concentration of 20 *μ*M.
