# Supplementary material for: Oxidative Stress Induced by the Deubiquitinase Inhibitor b-AP15 Is Associated with Mitochondrial Impairment
Source: Oxid Med Cell Longev. 2019 Jun 10;2019:1659468. doi: 10.1155/2019/1659468 (PMC6590552; doi:10.1155/2019/1659468)
Supplement: Supplementary Materials — Supplementary Fig. 1: induction of caspase cleavage activity by b-AP15 in HeLa cells. HeLa cells were exposed to 1 μM b-AP15 for 24 hours in the presence of absence of BIK siRNA or scrambled control siRNA as indicated. Exponentially growing HeLa cells were seeded in 100 mm dishes at 106 cells per plate and grown for 24 h and then transfected with BIK siRNA (Qiagen) at a final concentration of 10 nM using Lipofectamine RNAi MAX (Invitrogen). Cells were incubated for another 72 h and then treated with DMSO or b-AP15 for another 18 h. Apoptosis was determined by the measurement of the accumulation of caspase-cleaved apoptosis product in cells and culture medium using the M30 Apoptosense ELISA assay (VLVbio, Stockholm). The assay measures caspase-cleaved keratin 18 (K18-Asp396) (Bivén et al., Apoptosis 8 (2003) 263). BIK is a BH3-only protein that has been described to be associated with oxidative stress-induced apoptosis (Bodet et al., Br J Cancer 12 (2010) 1808). Means + S.D. are shown (n = 3). Statistical significance was determined using Student's t-test. Supplementary Fig. 2: oxidative stress induced by b-AP15 is not due to the inhibition of thioredoxin reductase (TrxR) activity. CB113 (ChemBridge ID 6943113), CB826 (ChemBridge ID 6556826), or CB916 (ChemBridge ID 6237916) was added to thioredoxin reductase enzyme assay mixes at 20 μM in Tris-EDTA and incubated for 5 hours. Reactions contained 50 nM rTrxR (21 U/mg), 200 μM NADPH, and 1 mg/mL BSA. Reactions were started by the addition of 2.5 mM of DTNB and absorbance was read at 412 nms. The TrxR inhibitor auranofin and Juglone were used as positive controls. Note, none of these three proteasome inhibitors targeted the TrxR activity at the concentration of 20 μM. [file 1659468.f1.pdf]

Supplementary Fig. 1

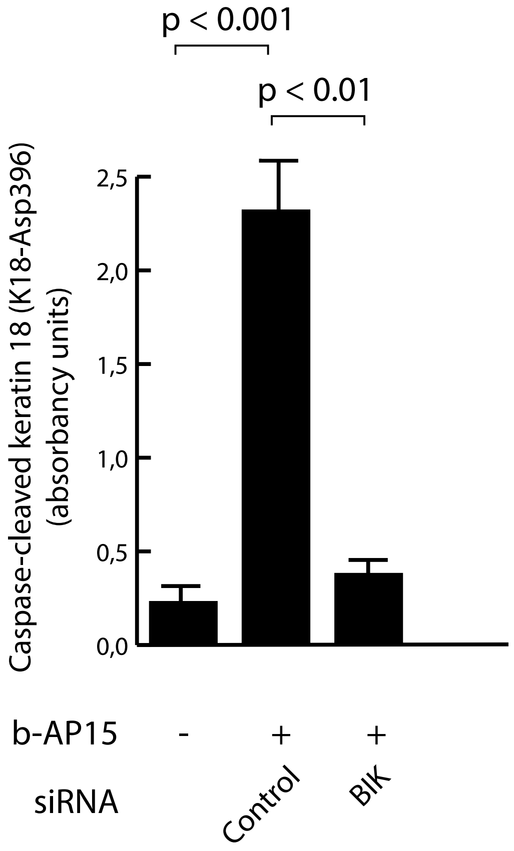

Induction of caspase cleavage activity by b-AP15 in HeLa cells.

HeLa cells were exposed to 1 μM b-AP15 for 24 hours in the presence of absence of BIK siRNA or scrambled control siRNA as indicated. Exponentially growing HeLa cells were seeded in 100 mm dishes at 10^6 cells per plate, grown for 24 h and then transfected with BIK siRNA (Qiagen) at a final concentration of 10 nM using Lipofectamine RNA_iMAX (Invitrogen). Cells were incubated for another 72 h and then treated with DMSO or b-AP15 for another 18 h. Apoptosis was determined by measurement of the accumulation of caspase-cleaved apoptosis product in cells and culture medium using the M30 Apoptosense ELISA assay (VLVBio, Stockholm). The assay measures caspase-cleaved keratin 18 (K18-Asp396) (Bivén et al., Apoptosis 8 (2003) 263). BIK is a BH3-only protein that has been described to be associated with oxidative stress-induced apoptosis (Bodet et al., Br J Cancer 12 (2010) 1808).

Shown are means ± S.D. (n = 3). Statistical significance was determined using Student's t-test.

Supplementary Figure 2

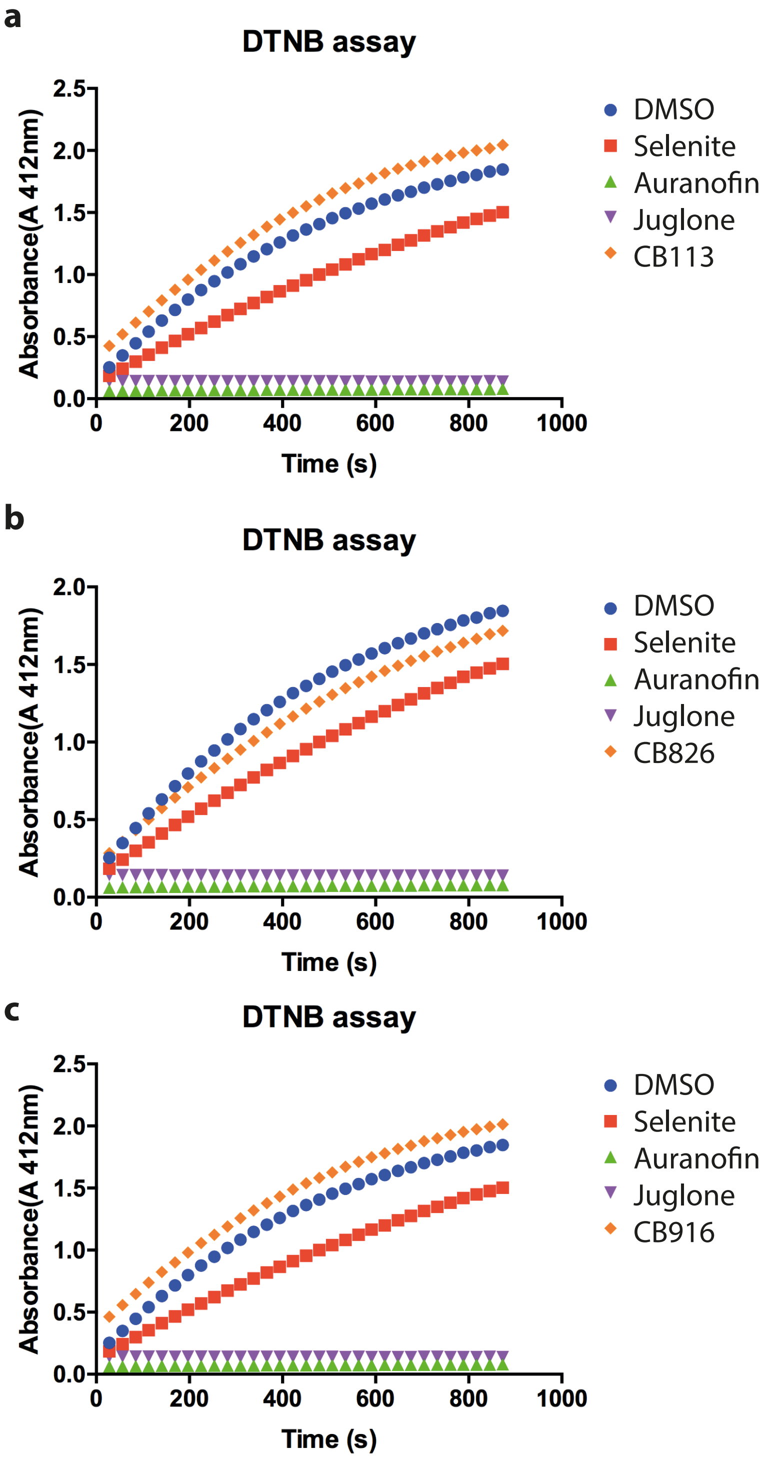

Supplementary Figure 2: Oxidative stress induced by b-AP15 is not due to the inhibition of thioredoxin reductase (TrxR) activity.

CB113 (ChemBridge ID 6943113), CB826 (ChemBridge ID 6556826) or CB916 (ChemBridge ID 6237916) were added to thioredoxin reductase enzyme assay mixes at 20 μM in Tris-EDTA and incubated for 5 hours. Reactions contained 50 nM rTrxR (21U/mg), 200 μM NADPH and 1mg/mL BSA. Reactions were started by addition of 2.5 mM of DTNB and absorbance was read at 412 nms. The TrxR inhibitor auranof-in and Juglone were used as positive controls. Note, none of these three proteasome inhibitors targeted the TrxR activity at the concentration of 20 μM.
